# “Even when people live just across the road…they won’t go”: Community health worker perspectives on incentivized delays to under-five care-seeking in urban slums of Kampala, Uganda

**DOI:** 10.1371/journal.pone.0244891

**Published:** 2021-03-26

**Authors:** Amy W. Blasini, Peter Waiswa, Phillip Wanduru, Lucky Amutuhaire, Cheryl A. Moyer

**Affiliations:** 1 University of Michigan Medical School, Ann Arbor, Michigan, United States of America; 2 Department of Health Policy, Planning, and Management, School of Public Health, Makerere University, Kampala, Uganda; 3 Department of Global Public Health, Karolinska Institutet, Solna, Sweden; 4 Department of Population Studies, School of Statistics and Planning, Makerere University, Kampala, Uganda; 5 Departments of Learning Health Sciences and OB/GYN, University of Michigan, Ann Arbor, Michigan, United States of America; UNC-Chapel Hill, UNITED STATES

## Abstract

**Background:**

Although under-five (U5) mortality in Uganda has dropped over the past two decades, rates in urban slum neighborhoods remain high. As part of a broader verbal and social autopsy study of U5 deaths, this study explored the perspectives of volunteer community health workers, called Village Health Teams (VHTs), on why children under five in Kampala’s informal settlements are still dying despite living in close proximity to nearby health facilities.

**Methods:**

This exploratory, qualitative study took place between January and March 2020 in the Rubaga division of Kampala, Uganda. VHTs from the slums of Kawaala and Nankulabye parishes, both located near a large government health center, were interviewed by a trained local interviewer to determine their perceptions of barriers to care-seeking and attribution for U5 childhood deaths. All interviews were audiotaped, transcribed into English, imported into NVivo V 12.0 and thematically analyzed using the Attride-Stirling framework.

**Results:**

20 VHTs were interviewed, yielding two global themes, the first focusing on VHTs perceptions of their role in the community to promote positive health outcomes, and the second focusing on VHTs’ perceptions of how prompt care-seeking is disincentivized. Within the latter theme, three inter-related sub-themes emerged: *disincentives for care-seeking at the health system level*, which can drive *negative beliefs held by families about the health system*, and in turn, drive *incentives for alternative health behaviors*, which manifest as “incentivized delays” to care-seeking.

**Discussion:**

This study illustrates VHT perspectives on the complex interactions between health system disincentives and the attitudes and behaviors of families with a sick child, as well as the reinforcing nature of these factors. Findings suggest a need for multi-pronged approaches that sensitize community members, engage community and health system leadership, and hold providers accountable for providing high-quality care. VHTs have enormous potential to foster improvement if given adequate resources, training, and support.

## Introduction

The number of children dying worldwide before their fifth birthday has dropped by half since 1990, from 93 deaths per 1,000 live births in 1990 to 39 in 2018 [1]. However, most child deaths are caused by conditions that are preventable or treatable with high-quality, cost-effective interventions [1]. The leading causes of death globally among children age 1 to 59 months are pneumonia, diarrhea, injuries and malaria [2]. Furthermore, the vast majority of under-five child mortality continues to disproportionately affect low- and middle-income countries. Notably, sub-Saharan African has the highest under-five mortality rate in the world [1]. To achieve the sustainable development target for under-five mortality of 25 or fewer deaths per 1,000 live births by 2030, there is an urgent need to improve childhood survival in high burden countries within Sub-Saharan Africa [3].

In Uganda, under-five mortality has declined from 151 deaths per 1,000 live births in 2000–01 to 46 deaths per 1,000 live births in 2018 [1]. This progress has been partially attributed to the government’s Childhood Survival Strategy, which aims to provide universal access to high-impact interventions, including micronutrient supplementation, malaria prevention and treatment, immunizations, prevention of mother-to-child transmission of HIV, and improved water and sanitation [4]. Nonetheless, improvements seen in aggregate, national-level data often mask sub-national, district, and local variability.

Up to two-thirds of Kampala’s urban population live in informal settlements, or ‘slums’ [5]. The living space is typically crowded, often with poor sanitation [6, 7]. Recent studies indicate that the burden of child deaths is higher among the urban poor compared to those in rural areas [8, 9]. Yet research regarding the barriers and potential solutions to reducing child mortality in Uganda has focused disproportionately on rural and peri-urban settings [10–14]. At the household level, barriers include illness recognition, decision-making to seek care, and financial costs [11, 12, 14]. At the facility level, issues around quality of care and timely referrals have been shown to be key barriers to improving child outcomes [11, 13, 14]. Yet childhood mortality in the urban areas of Uganda has not been adequately explored, particularly mortality among the urban poor who reside in informal settlements.

As part of a broader verbal and social autopsy study of under-five deaths in Kampala, this study aimed to explore the perspectives of volunteer community health workers, called Village Health Teams (VHTs), on why children under the age of five in two of Kampala’s informal settlements are still dying despite living in relatively close proximity to free government health facilities. VHTs working in the slums of Kampala function as a bridge between the health system and the community and are uniquely positioned to provide insight into potential challenges and opportunities to improve under-five mortality.

## Methods

### Study setting

This exploratory qualitative study took place between January and March 2020 in the Rubaga division of Kampala, Uganda, which is one of Kampala’s 5 divisions. Kampala is Uganda’s capital city with an estimated population of 1.5 million people [15], more than two-thirds of which reside in informal settlements [5]. This study focused on two parishes within the Rubaga division, Kawaala parish and Nankulabye parish. Both of these parishes predominantly contain informal settlements. Within Kawaala parish, there are 10 geographic zones and an approximate total slum population of 92,000 people [16]. Within Nankulabye parish, there are 9 geographic zones and an approximate slum population of 40,000 people [16]. In Kampala, healthcare is delivered through a combination of public, private and private not-for-profit health facilities. Kawaala and Nankulabye parish were purposively selected due to their proximal location to a public health facility, Kawaala Health Center IV. Their close proximity to a government-sponsored facility helps mitigate factors like cost and transportation when evaluating care-seeking behaviors of residents. Village Health Team members, known locally as “VHTs,” serve as voluntary community health workers and act as a bridge between the slum communities and the health center(s)/health system.

### Study participants

Participants included 20 VHTs currently working in the slums of Kawaaala and Nankulabye parish in the Rubaga division of Kampala, Uganda. VHTs included in the study were over the age of 18, actively practicing as a VHT for at least 3 months, and willing to participate in the study. Participating VHTs were purposively selected to represent the diverse geographical locations within informal settlements, including one VHT for each of the 11 zones of Kawaala parish and each of the 9 zones of Nankulabye parish. If thematic saturation had not been reached with the initial sample of 20, additional VHTs would have been recruited from the same catchment area. However, thematic saturation was reached, and additional interviews were not sought.

### Data collection

Approval was first sought from the parish coordinators to approach village health team members. A VHT from each zone was contacted by the study coordinator and asked if they would be interested in participating in a ‘key informant’ interview. Written informed consent was obtained before the start of each interview. Interviews followed a semi-structured interview guide that asked VHTs to describe what happens when a child under age five is ill, the barriers and facilitators to seeking healthcare for children under age five, and the role of the VHTs in care provision. Interviews were conducted in Luganda by a bilingual local research assistant trained in qualitative interviewing, and interviews typically lasted 30–60 minutes. The audio-recorded interviews were transcribed from Luganda into English, leaving intact any words or phrases that were particularly difficult to translate. All transcripts were reviewed for accuracy and completeness by the study team, which involved the lead researcher (AB) and the interviewer (LA) reviewing and discussing the transcript.

### Data analysis

All transcripts were read independently by two of the authors (CAM, AWB), who separately identified potential codes. The two separate code lists were discussed and combined into one initial coding schema that was then refined into a codebook with individual code descriptions and exclusion and inclusion criteria for each code. The codebook and all transcripts were uploaded into NVivo 12.0. Each transcript was coded by a primary coder, and 20 percent of transcripts selected at random were coded by a secondary coder. Any coding inconsistences were discussed, with subsequent revision and refinement of the codebook and coded transcripts. The ongoing iterative coding process ensured all team members were in agreement with regards to the data and codebook. Thematic analysis employed the Attride-Stirling framework [17].

### Ethical review

This study was reviewed and approved by the Makerere University School of Public Health Research and Ethics Committee, the University of Michigan Institutional Review Board, and the Uganda National Council for Science and Technology.

## Results

Table 1 illustrates the demographic characteristics of the participating VHTs. The VHTs ranged in age from 22 to 58 years old, with a mean age of 42.8 years. Fourteen out of the 20 respondents had at least 6 years of experience as a VHT, and most (11 out of 20) had less than a secondary school education. (See Table 1.).

**Table 1:** VHT demographics.

	n = 20
**Slum Parish**	
Kawaala	11/20
Nankulabye	9/20
**Experience (years)**	
1 to 5	6/20
6 to 10	8/20
11 +	6/20
**Age (years)**^1^	42.8 (22–58)
**Gender, Female**	11/20
**Highest Level of Education**	
Part or all of Primary (≤ 7 years)	2/20
Part of Secondary School (> 7 years, < 13 years)	9/20
Secondary (13 years)	2/20
Certificate, Diploma, Degree	7/20

^1^One VHT declined to provide their age.

In-depth interviews yielded two global themes, the first focusing on VHTs’ perceptions of their role in the community in promoting positive health outcomes, and the second focusing on VHTs’ perceptions of the many ways that prompt care-seeking is disincentivized, referred to as “incentivized delays.”

### Role of the VHT

When asked about their role as a VHT, participants listed a variety of duties: sensitizing the community before immunizations, administering immunizations, education of families about positive health practices, providing preventative measures such as bed nets, handing out deworming tablets, encouraging families to go to the health center for treatment, and arranging transportation when necessary.

“*Our role is to sensitize and visit people*, *to teach them how to handle young children*, *to take them to the hospital*, *to immunize them*, *and to keep a sanitary home*.”

(Kawaala, VHT4)

“*Ensuring that children in the community are immunized and sensitizing the residents about current disease outbreaks*. *We are also the link between the village and the health centers*, *though we do not really give out medicine*. *However*, *we do give out mosquito nets when they are supplied by the government*, *and we give out deworming tabs at schools*.”

(Kawaala, VHT11)

The VHT’s role in educating families and the broader community was a common theme.

“*Our biggest role is to educate and inform the mothers and children on how to maintain good health and sanitary conditions*.”

(Kawaala, VHT8)

“*We sensitize on family planning*, *immunizations*, *nutrition and general cleanliness*.”

(Nankulabye, VHT18)

Several VHTs highlighted the important role that they play in introducing immunization campaigns to the community, and how without this step, many people in the community would be unwilling to immunize their children.

“*My people will hardly allow their children to be immunized if I have not talked to them about it beforehand*.”

(Nankulabye, VHT20)

VHTs are also advocates on behalf of the health center and assist families in reaching care.

“*We help whoever we find that doesn’t have the means to transport themselves to the health center*, *and we bring them to the health center*. *They are treated and then we take them back home*.”

(Kawaala, VHT5)

The VHTs also highlighted outreach to the community, including on behalf of the local health center, as a key role. This function is particularly invaluable in times of disease outbreaks.

“*We have been organized into groups attached to Kawaala Health Center*. *In case of any outbreak*, *we are the link between the community and the hospital*. *We also give referrals*. *We have community meetings to inform the people on how to deal with different challenges*. *We are like representatives of Kawaala Health Center in our community*.”

(Nankulabye, VHT17)

### VHT perspectives on incentivized delays

**Fig 1:**
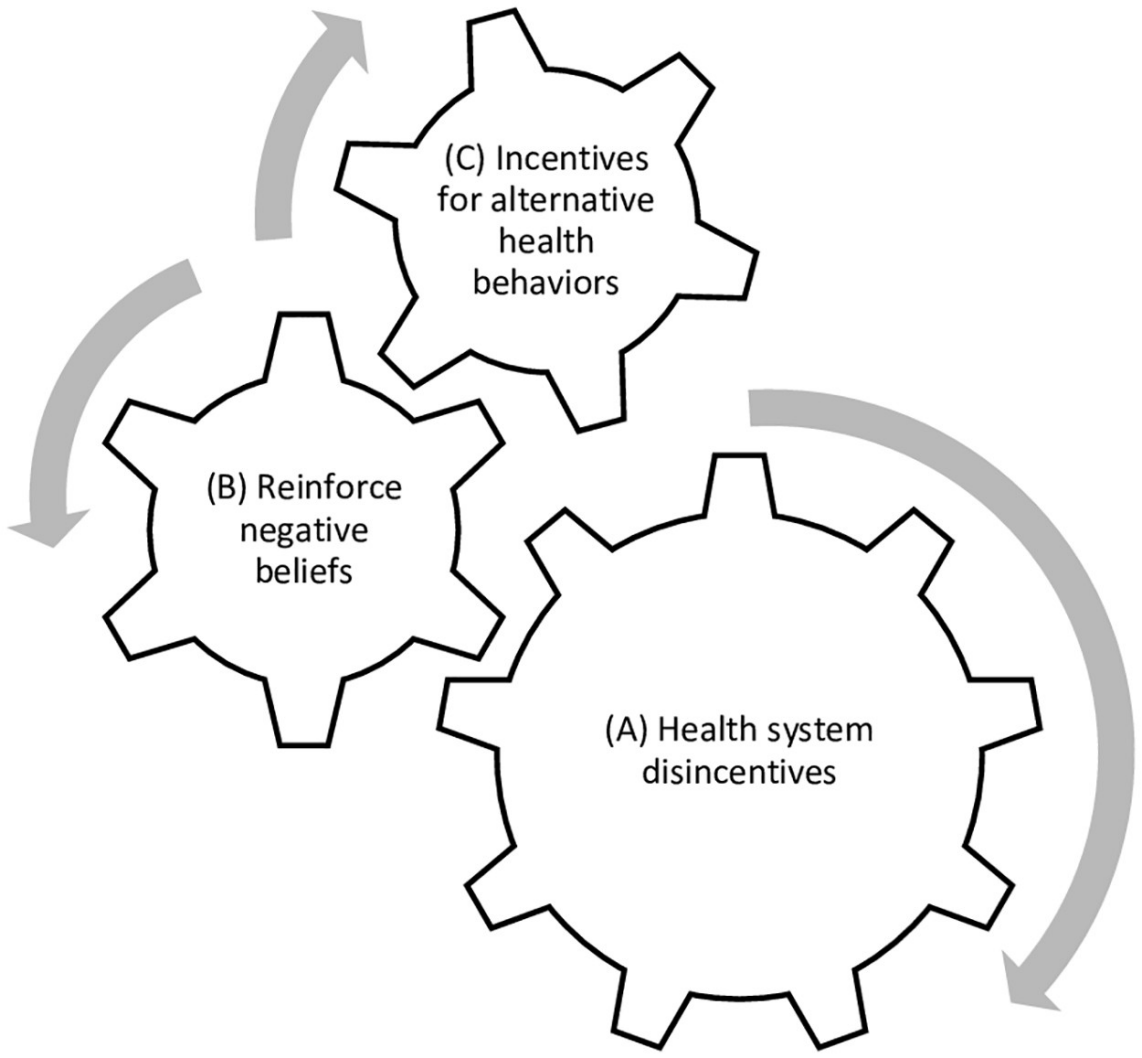
VHT perspectives on incentivized delays.

The second global theme that emerged from the data related to the many ways that prompt care-seeking is disincentivized in the informal settlements. Within this theme, three inter-related sub-themes emerged: *disincentives for care-seeking at the health system level*, which can drive *negative beliefs held by families about the health system*, and in turn drive *incentives for alternative health behaviors*, which manifest as “incentivized delays” to care-seeking. This is illustrated in Fig 1, explaining from the VHTs’ perspectives the interrelated and reinforcing mechanisms that incentivize delayed care-seeking.

Fig 1 illustrates the sub-themes as a series of cogs (or gears). The first cog symbolizes disincentives at the health system level, which can then drive the second cog, negative beliefs held among families about the health system. Ultimately, both of the previous cogs can drive the third cog, incentives for alternative health behaviors that can manifest as “incentivized delays” to seeking care at the health center.

### Health system level disincentives

As the largest cog in Fig 1 illustrates (A), VHTs described multiple disincentives to care-seeking for childhood illness that are built into the health system, including delays from standing in long lines, unprofessional behavior by medical professionals, high cost of medicine, lack of medicine available at the health centers, and extra payments (bribes) to medical professionals in exchange for care. VHTs across both parishes described such factors as significantly impacting families’ willingness to go to the health center.

#### Long lines/delays at health center

The need to wait in long queues at the health center was cited by many respondents as a significant barrier to care-seeking, especially when the wait did not appear to be worthwhile.

“*…people give the excuse that they will spend the whole day in line*. *This is a factor that causes people to be lazy about bringing their child to the health center because they feel like a whole day spent at the health center will be a wasted day*.”

(Kawaala, VHT4)

“*Firstly*, *this place is too congested*. *So people wait for a long time and get tired*. *Secondly*, *their friends discourage them after they have been there and not received any medicine*.”

(Nankulabye, VHT17)

The long waits are especially problematic for women and families with competing responsibilities.

“*The issue is that most of the single mothers don’t have anyone to leave in charge of their small stalls (businesses) when they go to wait in the long lines at the hospital*.”

(Kawaala, VHT1)

“*… Spending time at the hospital… requires them to neglect all their other daily activities and accept waiting in hospital lines*. *So the delays at the hospital are a discouragement to the parents*.”

(Kawaala, VHT1)

#### Unprofessional behavior by medical professionals

In addition to long lines, VHTs described ‘harsh doctors’ and mistreatment experienced by families seeking care, which can disincentivize future care-seeking. Mistreatment was described as being shouted at, taunted, or neglected.

“*People dislike going to government hospitals because of the way the nurses treat the patients*. *… They shout at people*, *neglect them*, *and delay to work on them*.”

(Kawaala, VHT7)

“*Well*, *some people [don’t go to the health center] out of fear because people there just shout at you*.”

(Nankulabye, VHT15)

“*… Hospitals are hugely lacking in empathy and religion in their hearts and this is a huge factor in why people feel discouraged from coming to the health facilities*. *[Health providers] taunt the people …(and say that) they don’t deserve any special attention*. *This harshness doesn’t help the situation*.”

(Kawaala, VHT5)

#### Cost of medicine

VHTs described the cost of medicine as a significant barrier for families with sick children, since, as one VHT said, “Most parents are poor and do not have the money to buy medicine.” (Kawaala, Ahmed, Environment).

“…*When someone comes to the health center more than once and never receives free medicine*, *all they receive are prescriptions*, *then they get discouraged*.”

(Nankulabye, VHT20)

“*Even when they say government hospitals have free medicines*, *it’s hard to access these services*.”

(Nankulabye, VHT19)

VHTs described families waiting in long queues with a sick child, only to be told that they needed to pay for medication that was far beyond what they could afford.

#### No medications

In addition, VHTs described facilities often running out of the necessary medications or not having them on site. The lack of available medications was cited repeatedly as a deterrent to care-seeking.

“*…These hospitals lack a supply of medicines*, *so the parents are required to buy them from elsewhere*.”

(Kawaala, VHT1)

“*… sometimes they get there [to the health center] and there are no medicines*. *This discourages them*.”

(Nankulabye, VHT17)

“*I would rather take my child to a [lower-level] clinic than go to Kawaala because even when you go there*, *there is no medicine*.”

(Kawaala, VHT3)

#### Bribes

Another factor that VHTs indicated can serve as a disincentive to care is the expectation for families to “contribute to the maintenance of the health center”, which is typically code for a bribe paid to the provider. This adds to the unanticipated costs of seeking care at a health center.

“*…These people give check-ups and tell you to provide some little money which is meant to maintain the health center*.”

(Nankulabye, VHT14)

“*The nurse will just tell the family that they need money for different stuff*. *The government actually provides things*, *like gloves*, *cotton and all*, *so you will have to pay money for them*. *If you don’t have the money*, *she (the nurse) will not touch you at all*.”

(Nankulabye, VHT19)

### Reinforce negative beliefs

The second cog in Fig 1B illustrates that VHTs perceive health system disincentives (Cog A) can drive and reinforce negative beliefs among family members with regard to seeking care at a health facility (Cog B). Several VHTs commented on how the health system disincentives reinforced negative beliefs held among community members about the health system more broadly, as well as about the government (Fig 1B). VHTs commented on the perspective, or “mentalities,” of families as barriers or inhibitors to seeking care at the hospital.

“*But here*, *even when people live just across the road from the health facility*, *they won’t go due to their cultural beliefs about the health center*.”

(Kawaala, VHT9)

“*It is usually the image people have about the health centers*.”

(Nankulabye, VHT14)

“*It is just the wrong mentality*.”

(Kawaala, VHT9)

More specifically, the deterrents at the health system level were noted to contribute to distrust in the health system and the government. VHTs described stories being passed from one family to another within the slum communities, helping to develop communal knowledge and beliefs that reinforce distrust of the health system.

“*So*, *when one goes to a health center and they aren’t treated but are only given Panadol*, *the rest get discouraged upon hearing what happened to the person who went*.”

(Kawaala, VHT5)

“*Secondly*, *their friends discourage them after they have been there and not received any medicine*.”

(Nankulabye, VHT17)

“*… There is the stereotype in these people’s mind that these government facilities don’t work*.”

(Kawaala, VHT3)

“*Most people don’t trust government medicine*.*”*

(Nankulabye, VHT19)

Several VHTs indicated that much of this distrust is rooted in a skepticism that the services a family expects to receive will not be provided at the public health center. While the government is supposed to provide free healthcare, the people who go to the public health facilities typically incur unanticipated costs. If the family cannot afford these costs, then the help they receive is limited.

“*Most people just don’t trust the government because they think nothing is free at a health center*.”

(Nankulabye, VHT19)

“*…they don’t trust that the government provides actually free medical care*.”

(Nankulabye, VHT19)

“*So*, *they even say that there is no difference between government and private facilities because even here*, *if you don’t have money*, *the doctors don’t really work on you*.”

(Kawaala, VHT3)

VHTs also commented on how families from the slums are especially vulnerable to the negative consequences of unprofessional behavior by health center staff. The systemic issue of “harsh doctors” reinforces the perspective that going to the health center means one is made to feel inferior and worthless. The fear of harsh treatment and judgement by medical staff heavily discourages families from seeking care.

“*So sometimes even the handling of these people by the medical professionals matters*. *Don’t be fooled*, *these poor people can’t come to health facilities but when they come*, *they want to be cared for because their child is sick and these health professionals keep yelling at them not to stand there*, *or do this or that and telling them how they aren’t the first person to get sick and others are dead*. *Even the cleaners bark at these people so this discourages people because they come here to be cared for*, *not to be made to feel worthless*.”

(Kawaala, VHT5)

“*Besides*, *most of them feel so inferior and lack self-esteem*, *so they find it difficult to go to the hospital*.”

(Nankulabye, VHT14)

The distrust of the government and health system extends beyond seeking care at the health center. The VHTs commented on how such a culture of distrust also makes preventative health efforts, such as immunizations and mosquito nets, difficult to implement in the community.

“*We have a lot of challenges; for example*, *during immunizations*, *we have to strongly persuade parents because they think we are going to kill their children*.”

(Nankualbye, VHT16)

“*In the recent exercise where we immunized children*, *many people often hid their children*. *We later found that they never really immunized their children and they don’t support the government’s plans*.*”*

(Kawaala, VHT10)

### Alternative behaviors

The third cog of Fig 1C illustrates VHTs perceptions that disincentives at the health system level (Cog A) reinforce negative beliefs about the health system (and government) (Cog B), which may ultimately drive the family of a sick child to pursue alternative health behaviors (Cog C), such as herbal/traditional medicine, self-medication, and symptom management from local clinics. Only when the child’s symptoms are most severe does the family seek help from the health center.

“*A parent can decide to pick leaves [local herbs] and smear them on the child or use left-over tablets from previous treatments without knowing the current problem affecting the child*, *and in the end*, *the illness just worsens*.”

(Kawaala, VHT2)

“*Sometimes*, *they advise each other to first try out the herbal treatments … so they try to treat the children themselves*.”

(Kawaala, VHT8)

“*…Some of them stupidly act with recklessness and decide to revert to herbs and other things they claim to have got from their ancestors*.”

(Kawaala, VHT10)

Many VHTs cited the high cost of drugs prescribed at the health center and the lack of drugs at the health center as reasons for families to pursue herbal treatments first, suggesting that Cog A (health system disincentives) is also interacting with Cog C (alternative health behaviors).

“*In fact*, *it’s not a thought but it’s the truth*. *In most cases*, *there are no medicines at the health centers*, *so parents end up using herbal medicine because they can’t afford to buy the medicines prescribed for them at the health centers*.”

(Nankulabye, VHT18)

“…*They turn to herbal treatments from wherever they might have heard of any concoctions that might heal the child because they can’t afford the medicine that was prescribed*.”

(Kawaala, VHT5)

“*It [herbal medicine] is actually common*, *because people are afraid of hospital costs*.”

(Nankulabye, VHT14)

Self-medication, with left-over prescription pills or a pain killer like Panadol (acetaminophen), are common first methods of treatment. In part, this method is employed to delay seeking care from the health center.

“*Each parent does it differently*, *but most of them first give self-medication*. *They buy some paracetamol*, *and when it fails*, *they rush to the hospital*.”

(Nankulabye, VHT19)

“*These people first treat themselves*. *They want to buy Panadol and administer it themselves*. *Also*, *the lack of money is a big factor in this*.”

(Kawaala, VHT6)

“*Most parents first practice self-medication*. *They buy tabs from the clinics*.”

(Nankulabye, VHT20)

Additionally, the VHTs pointed out that families commonly go to their local clinic to seek a faster and cheaper treatment for their children than going to the higher-level government health center. Local clinics take many forms, ranging from drug shops to a clinic staffed with a provider. Similar to herbal treatments and self-medication, local clinics are another stop-gap to delay visiting a health center.

“*They usually first go to clinics*, *they like going to clinics because they pay very little money there*. *They come to the hospital when it is their last option and the child is dying*.”

(Kawaala, VHT11)

“*Most of the time*, *when children get sick*, *they first run to the nearby clinics*. *One will get a thousand shillings*, *go to the clinic*, *and say the child is burning with a fever*, *which prompts the people there to simply administer a pain killer and the sickness worsens*.”

(Kawaala, VHT3)

“*They often send the older sibling with the child to get some drugs from the drug shop*. *They also won’t think to bring that child to the health center*, *which causes the child to die*.”

(Kawaala, VHT4)

VHTs described how when all alternative health treatments have failed, the family will decide to take the child to the health center. Many VHTs cited worsening symptoms as a reason for seeking care from the health center.

“*If the condition doesn’t improve*, *the father returns at night and dictates the kind of medicine to buy from the clinic*. *This is because they don’t have money to take the child to the hospital*. *It is when the condition completely fails to improve that they take the child to the hospital*.”

(Kawaala, VHT7)

“*They come to the hospital when it is their last option and the child is dying*.”

(Kawaala, VHT11)

### Inter-relationship

Alternative health behaviors can be seen as “incentivized delays” in care-seeking due to disincentives at the health system level. If the family only reaches the hospital when the child is in critical condition, this can further stress the health system, as illustrated in the following quote. This added burden gives energy back to at least one of the health system level disincentives, the unprofessional behavior of healthcare staff.

“*Others just want to come to the health center and then have their issues worked on ahead of everyone else they found at the facility*. *Even when they haven’t come early*, *they want to be given priority over the others*, *and this sometimes vexes the health workers who respond in a rather harsh manner and this is the basis for why people claim that the health workers are harsh to them*.”

(Kawaala, VHT8)

“*…People also need to be sensitized out of that notion that they won’t be worked on if they don’t have money because this leads to people not taking their children to the hospital and before you know it*, *they have lost the child*.”

(Kawaala, VHT3)

### VHT-generated countermeasures

While discussing opportunities to improve the situation for children under age five living in the slums of Kampala, the VHTs called for strengthening the role of the VHT in the urban slum community. Both willing and well-positioned, VHTs see the opportunity to leverage the existing community health worker network and extend their role in family education and first aid, and even serve as patient navigators within the health system.

“*VHTs need more trainings on how to help improve their communities and how to educate mothers on caring for their children*.”

(Nankulabye, VHT13)

“*It would help if we as VHTs received trainings on how to give the first treatment*.”

(Nankulabye, VHT15)

“*…a program like Mengo hospital’s training could really help equip VHTs with the necessary skillset required to help out other professional workers to quickly offer help to these children*. *This would cost a bit of money though*.”

(Kawaala, VHT8)

“*We could have VHTs in the hospitals so that those with esteem issues are welcomed*.”

(Nankulabye, VHT14)

## Discussion

This study illustrates VHT perspectives on the complex interactions between health system disincentives and the attitudes and behaviors of families with a sick child, as well as the reinforcing nature of these factors. According to embedded community health volunteers in urban slum settings, factors at the health system level reinforce negative beliefs among families about the health system and reinforce less-than-ideal care-seeking behaviors. VHTs described health system-level disincentives such as long lines at the health center, unprofessional behavior of healthcare staff, high cost of medicine, lack of medications at the health center, and bribes to health facility staff. VHTs indicated that these experiences in turn reinforced negative beliefs among families, including distrust of the health system, skepticism toward services provided at the health center, and being made to feel worthless or inferior at the health center. The combination of health system disincentives and negative beliefs can lead to alternative health behaviors, also called “incentivized delays” to reaching the health center, including using herbal/traditional medicine, practicing self-medication, visiting local clinics that are staffed with variably trained providers, and waiting until symptoms are severe before going to the health center. The incentivized delays can in turn reinforce health system disincentives. For example, arriving at the health center only when symptoms are severe can add to the work burden of the health providers and fuel the perceived unprofessional behavior by healthcare staff. Nonetheless, VHTs believe that their unique position at the intersection between the community and the formal health sector can help bridge the gap to improve under-five outcomes.

This study describes what we have termed “incentivized delays” to care-seeking. Delayed care-seeking is a well-documented factor associated with under-five child mortality [10, 14, 18]. Other researchers have documented contributors to delayed care-seeking such as geographic barriers, financial barriers, household decision-making, poor perceptions of care, inadequate facility supplies and staffing, lack of health care worker professionalism, and unfamiliarity with danger signs [14, 19–21]. In our study, we explored care-seeking in a context where the health facility was in close proximity (mitigating geographic barriers) and care was theoretically free (mitigating financial barriers). While we found similar barriers to those described in previous research, we found one of the most important additional concerns related to distrust of the health system. Distrust of the healthcare system stemmed from multiple factors, which included but was not limited to unprofessional behavior of healthcare staff, lack of medications that were expected to be provided by the health center, and bribes to health facility staff.

This finding aligns with previous research linking perceived corruption, specifically the need to pay bribes within the healthcare setting, with reduced care-seeking in 32 countries in sub-Saharan Africa [22]. Interestingly, social networks have proven helpful in navigating or mitigating such barriers [23]: if patients know that access is challenging, they may mobilize financial resources or direct connections to the health system to help gain prioritized access. Given the strong ties between VHTs and the formal health sector, it is possible that VHTs could serve to boost families’ ‘social capital’ and ensure they receive high quality care.

The issue of quality of care is another aspect of these findings that warrant further attention. The World Health Organization has articulated standards of care and quality for maternal and newborn health provided in facilities, including both the technical and interpersonal aspects of care [24]. Standard #5, as articulated by the WHO, is that women and newborns are to “receive care with respect and preservation of their dignity” [24]. Studies to improve the interpersonal aspects of maternal care have shown that training- and education-based interventions have increased women’s experiences of respectful care and reduced disrespect and abuse in provider-patient interactions [25–28]. Our findings reiterate the need to train for, measure and hold facilities accountable to these WHO standards.

This study has significant implications for efforts aimed at reducing under-five mortality in slum neighborhoods. The first is the need for multi-pronged approaches. Efforts to sensitize community members to increase care-seeking at local facilities are likely to fall short when their experiences at those facilities reinforce negative beliefs and deter care-seeking. At the same time, intervening at the health system level requires significant engagement at the government level (ensuring funding and supply chain viability), from hospital leadership (ensuring staffing and accountability), and from individual providers (ensuring high quality, respectful care provision). Only when efforts triangulate will meaningful change occur. The second important implication is the recognition that care-seeking is nested within a broader cultural context, and in this case, distrust of the health system and the broader government are not trivial barriers to overcome. Efforts that genuinely address community members’ feelings of marginalization are a necessary precursor to efforts to improve care-seeking at government-run facilities.

As the VHTs themselves brought to light, the urban volunteer community health worker is an existing bridge between the community and the health system. VHTs have enormous potential to help mitigate community members’ feelings of marginalization, and they may also be able to improve community members’ experiences within the health system by playing the role of a “patient navigator,” or someone who can help community members get the care they need, even serving as an advocate on their behalf when necessary.

This study has several strengths, most notably that it explores the unique perspectives of village health team members, who are well-positioned to understand the landscape of the health system as well as the perspectives of mothers and families with ill children in the community. VHTs hold important insight into the beliefs and thought process of their community members. Another strength is that the study explicitly focuses on slum communities and the VHTs who work and serve urban informal settlements. This is a population that is largely understudied, especially with regard to barriers to care-seeking. Furthermore, when VHTs describe the facilitators and barriers to the health of children under age five in the community, distance is not a factor due to the close proximity of slums to a public health center. One of the study limitations is that the qualitative approach does not allow for statistical generalizations of the findings. Additionally, the interviews were conducted in the local language and translated into English for analysis, which could allow for subtle differences in meaning to be lost in translation.

As outlined in the Reproductive, Maternal, Newborn, Child and Adolescent Health (RMNCAH) Sharpened Plan for Uganda [29], there is an urgent need to increase access to care for high burden populations and to understand and address system bottlenecks. This study illustrates some of the challenges and opportunities in two urban slums in Kampala City, providing both a novel conceptual framework to explain the interrelated and mutually-reinforcing disincentives to care (“incentivized delays”) as well as potential pathways to expand the reach of VHTs to bridge the gap between communities and high-quality care provision. Future research and programmatic efforts are warranted to explore mechanisms to incentivize providers to be more responsive to the needs of the poor, such as results-based financing, leveraging public- and private-sector partnerships, and developing a public healthcare strategy that suits the diverse needs of the urban poor while empowering providers with the tools to offer high-quality care. Furthermore, this raises the question whether results-based financing and public-private partnerships are viable possibilities for incentivizing providers to be more responsive to the needs of the poor. Given the recalcitrance of under-5 mortality in urban slums, it is clear that there is a need for new models of care if change is ever to be seen.
